# Control of the tomato leaf miner, *Tuta absoluta* (Meyrick) (Lepidoptera: Gelechiidae) larvae in laboratory using entomopathogenic nematodes from subtropical environment

**DOI:** 10.21307/jofnem-2020-013

**Published:** 2020-03-06

**Authors:** Bonginkhosi E. Dlamini, Nelisiwe Dlamini, Michael T. Masarirambi, Nxumalo Kwanele A.

**Affiliations:** 1 Department of Crop Production, Faculty of Agriculture, University of Eswatini, P.O. Luyengo, M205, Manzini, Kingdom of Eswatini; 2 Department of Horticulture, Faculty of Agriculture, University of Eswatini, P.O. Luyengo, M205, Manzini, Kingdom of Eswatini

**Keywords:** Biological control, *S. jeffreyense*, *S. yirgalemense*, Tomato, *Tuta absoluta*

## Abstract

Tomato (*Solanum esculentum*) is one of the vegetable crops grown by both smallholder and commercial farmers in the Kingdom of Eswatini. Tomato leaf miner, *Tuta absoluta* M. is a major insect pest of tomatoes resulting in reduced tomato yields throughout the country. The study investigated the virulence of two sub-tropical EPN species on *T. absoluta* larvae. *Steinernema yirgalemense* and *S. jeffreyense* at different concentrations (0, 20, 40, 60 IJs/insect) were screened for efficacy (i.e. mortality rate) against larvae of *T. absoluta* in laboratory bioassays. Results obtained showed that *S. yirgalemense* and *S. jeffreyense* were able to kill *T. absoluta* larvae without significant differences between the two EPN species in 24-well bioassay trays. Significantly higher (*p* < 0.05) mortality was observed when 60 IJs/insect was used. The combination of *S. yirgalemense* at 60 IJs/insect (100%) and *S. jeffreyense* at 60 IJs/insect (98.3%) resulted in significantly higher (*p* < 0.05) mortality, compared with the other four combinations of EPN and concentration tested. In the leaf bioassays, *S. yirgalemense* (58.8%) resulted in significantly higher (*p* < 0.05) mean mortality compared to *S. jeffreyense* (46.4%). *Steinernema yirgalemense* at 60 IJs/insect resulted in significantly higher mean mortality compared to the other EPN and concentration combinations in the leaf bioassay. The results indicated that both EPNs tested were effective against *T. absoluta* larvae. *Steinernema yirgalemense* at 60 IJs/insect can effectively find *T. absoluta* larvae inside leaf mines, but large-scale field trials are recommended to demonstrate the potential use of the biocontrol agent within integrated pest management programs.

Tomato plants are among the world’s most cultivated crops and they are cultivated by both smallholder and commercial farmers in the Kingdom of Eswatini (FAO, 2012). Tomatoes are targeted by a vast number of insect pests and diseases including bacterial wilt (*Ralstonia solanacearum*), fusarium wilt (*Fusarium oxysporum*) and tomato leaf miner, *Tuta absoluta* (Lepidoptera: Gelechiidae). Tomato leaf miner is one of the most consequential lepidopteran pests that affect tomato plants (Husin, 2017). Silva et al. (1998) reported that *T. absoluta* is a limiting factor for tomato production throughout the world, resulting in 70% losses in most tomato growing areas. The insect pest is able to attack fresh tomatoes and those grown for processing (Desneux et al., 2011). The newly introduced lepidopteran pest from South America has high reproduction potential; capable of producing 10 to 12 generations per year under the favorable conditions (Derbalah et al., 2012). With such high reproduction potential, they are likely to undergo genetic changes (mutation) which in turn leads to the development of pesticide resistant populations (Husin, 2017).

Tomatoes are attacked by *T. absoluta* at any developmental stage and the primary method for managing *T. absoluta* involves the use of synthetic insecticides (Tropea et al., 2012). The use of synthetic chemicals is not only detrimental to the environment and human health, but has other serious disadvantages, including reduced profits due to high insecticides cost, destruction of natural enemy populations, build-up of insecticide residue on the tomatoes and insect resistance (Lietti et al., 2005; Husin, 2017). The efficiency of chemical control on *T. absoluta* infestations has been reported to be poor due to the entophytic habit of the larvae, which are protected in the leaf mesophyll or inside fruits (Mallia, 2009; Terzidis et al., 2014). Herbert et al. (2001) reported that higher levels of resistance to Abamectin, Aartap and Permethrin were correlated with greater use of these compounds by tomato growers in Brazil. The finding suggested that the variation in insecticide use in Brazil resulted in variation in the susceptibility of *T. absoluta* (Herbert et al., 2001).

The use of environmentally sound insect pest management strategies is important to minimum use of insecticides in tomato fields. Environmentally friendly strategies include cultural control (e.g. crop rotation, selective removal and destruction of infested plant materials), and the use of natural enemies (parasitoids, predators and entomopathogens) (Husin, 2017). Entomopathogenic nematodes (EPNs) are good alternatives to synthetic insecticides, are soil-dwelling organisms that attack insect pests that live in, on, or near the soil surface and can be used effectively to control important insect pests (Adams and Nguyen, 2002). EPNs in the families Steinernematidae and Heterorhabditidae do not affect non-target species, do not leave residues (Georgis et al., 2006) and are essential biocontrol agents used for controlling insect pests (Grewal and Georgis, 1999).

These EPNs are capable of penetrating and killing their hosts within 24–48 h of nematode invasion, which is caused by their mutualistic relationship with bacteria and of the genera *Photorhabdus* and *Xenorhabdus* that are carried in the intestine of *Heterorhabditidae* and *Steinernematidae*, respectively (Akhurst and Boemare, 1990; Husin, 2017). EPNs were reported to control *T. absoluta* but the use of sub-tropical EPN species is not yet known to farmers in the Kingdom of Eswatini. In this study, we tested the virulence of two sub-tropical EPN species on *T. absoluta* larvae.

## Materials and methods

### Source of insects

*Tuta absoluta* larvae were collected from infested tomato fields around Mankayane (26 °44’58”S 31°02’56”E), kept in Perspex boxes measuring 15 × 20 cm and transported to the Entomology laboratory, Faculty of Agriculture, University of Eswatini. *Tuta absoluta* was mass-reared in rearing cages (50 × 50 × 50 cm) (Vermandel, Hulst, The Netherlands) on tomato plants at 25 ± 2 °C, 65 ± 5% RH, with a 16:8 L:D photoperiod. Rearing cages (60 × 60 × 90 cm) (Vermandel) with tomato plants on pots were used to mass-rear adult *T. absoluta* and were allowed to lay eggs for 48 h. The tomato plants with eggs were transferred back to the other rearing cages measuring 50 × 50 × 50 cm. After hatching, larvae were allowed to feed on potted tomato plants at *ad libitum*. Data on the developmental time and morphological descriptions was used to separate between the four *T. absoluta* instars (Cuthbertson et al., 2013; Vargas, 1970). Last instar larvae were then harvested by means of opening the mines and picking the larvae. *Tenebrio molitor* (Coleoptera: Tenebrionidae) (mealworm) larvae, used for culturing nematodes, were reared on a diet comprised of wheat bran, as reported by Van Zyl and Malan (2013).

### Source of nematodes

Two laboratory reared EPN species, *Steinernema yirgalemense* Nguyen, Tesfamariam, Gozel, Gaugler & Adams 2004 and *S. jeffreyense* Malan, Knoetze & Tiedt 2015 were sourced from the EPN collection of the Nematology Laboratory, Department of Conservation Ecology and Entomology, Stellenbosch University, South Africa. The IJ rearing and harvesting procedures were carried out according to the methods presented by Kaya and Stock (1997), using mealworm larvae kept at room temperature (±25°C). The IJs were harvested from the White trap during the first week of emergence, stored horizontally using 500-ml vented culture flasks, and used within one month after harvesting. The culture flasks were shaken on a weekly basis, to increase the amount of aeration, and the survival of the IJs, during storage. The origin of the two EPN species used in the study is indicated in Table 1.

**Table 1.** List and characteristics of the *Steinernema* species used in the study.

Species name	Strain	Habitat	Locality	GenBank accession number	Length of IJ (μm)	Body width of IJ (μm)	Reference
*S. yirgalemense*	157-C	Citrus orchard	Friedenheim, Mpumalanga	EU625295	685 (570–740)	29 (24–33)	Malan et al. (2011)
*S. jeffreyense*	J194	Guava tree	Jeffrey’s Bay, Eastern Cape	KC897093	924 (784–1043)	35 (23–43)	Malan et al. (2016)

### Laboratory bioassay

Virulence experiments were conducted in 24-well bioassay plates (flat-bottom, Nunce, Cat. No.144530, Thermo Fisher Scientific (Pty) Ltd, Gauteng, Johannesburg, South Africa). The 24-well bioassay protocol was used to test the potential of *Steinernema yirgalemense* and *S. jeffreyense* to infect *T. absoluta* larvae under optimal laboratory conditions. Alternate wells were lined with a circular piece of 13-mm-diameter filter paper, to secure an even distribution throughout the well. Each alternate bioassay well with a *T. absoluta* larva (*n* = 60), was inoculated with 20, 40, 60 IJ/50 μl water, while the control received distilled water only. Each piece of filter paper was inoculated with a predetermined concentration (Navon and Ascher, 2000) of IJs in filtered tap water. A control treatment comprised of 50 μl filtered tap water. One insect was added to each alternate well, and the trays were closed with the lid, placed in a closed plastic container lined with moistened tissue paper, and left in a growth chamber at 25°C for 48 h. For each treatment, five trays with 12 wells were used (*n* = 60). Mortality by nematode infection was confirmed after 48 h, by dissection under a stereo microscope to check for the presence of EPNs. The procedure was repeated on a separate date, with a different batch of nematodes.

### Leaf trial

The experimental design consisted of eight tomato leaves for each treatment which was replicated four times in a completely randomized design. *Tuta absoluta* larvae were removed from infested tomato leaves collected from the field with the help of a needle and a camel hair brush under a light microscope. In total, 12 *T. absoluta* larvae were placed on the upper side of fresh tomato leaves and each leaf was placed in a petri dish. The lid of each petri dish was sealed with Parafilm (Pechiney Plastic Packaging, Neenah, WI, USA) to prevent *T. absoluta* larvae from escaping. The leaves containing the larvae were left for 24 h to mine and develop well-formed mines. *Steinernema yirgalemense* and *S. jeffreyense* at different concentrations (20, 40, 60 IJs/insect) were prepared and sprayed on the top side of the infested leaves, while the control treatments received distilled water only. Petri dishes, with the sprayed tomato leaves, were then placed in one plastic container per treatment, lined with wet paper towels to ensure high humidity, and kept in an incubator at 25°C. *Tuta absoluta* larval mortality was checked after 2 days and mortality was confirmed by dissecting the dead larvae under a light microscope to determine whether EPNs are present inside the dead larvae. The procedure was repeated on a separate date, with a different batch of EPNs to confirm results. Total numbers of dead larvae per EPN species at different EPN concentration were recorded.

### Data analysis

Virulence assays were analyzed using analysis of variance (ANOVA); if the *F*-value was significant (*p* < 0.05), the means were differentiated by LSD MEANS (SAS Institute, 1985). The mortality data were corrected for the corresponding control mortality, using the formula: CM (%) = {(T−C)/(100−C)} × 100, where CM is the corrected mortality, T is the percent mortality in treated insects, and C is the percent mortality in untreated insects (Abbott, 1925). If no significant interactions were found to occur between the main effect of the test dates and the treatments concerned, data obtained from the two test dates was pooled and analyzed, using a two-way ANOVA.

## Results

### Laboratory trial

Analysis using a one-way ANOVA showed no significant effect (*F*_(3, 36)_ = 26.34, *p* < 0.001) of the treatment on percentage mortality. *Steinernema jeffreyense* and *S. yirgalemense* resulted in of 93.0 and 93.6% *T. absoluta* larval mortality, respectively. The EPN concentrations gave 6.5, 87.1, 94.6 and 99.2% mortality for 0, 20, 40 and 60 IJs/insect, respectively. All the four EPN concentrations resulted in *T. absoluta* larval mortality that was significantly different (*p* < 0.05) from each other.

**Figure 1:**
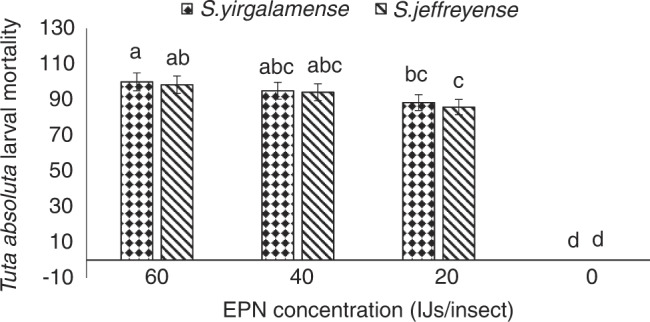
Mean percentage mortality (95% confidence level) of *Tuta absoluta* larvae inoculated with 0, 10, 20, and 40 IJs/insect of *Steinernema yirgalemense* and *Steinernema jeffreyense* (two-way ANOVA: *F*_(3, 28)_ = 17.50, *p* < 0.001) in a laboratory trial. Different letters above the vertical bars indicate significant differences (*p* < 0.05).

The mortality of *T. absoluta* larvae in the laboratory trial ranged from 0 to 100% after inoculation with 0, 20, 40, and 60 IJs/insect over a period of 2 days, with significant effect (*F*_(3, 28)_ = 17.50, *p* < 0.001) of the treatment on the percentage mortality. *Steinernema yirgalemense*, at 60 IJs/insect (100%), provided significantly higher (*p* < 0.05) mortality of the *T. absoluta* larvae, than *S. yirgalemense* at 20 IJs/insect, *S. jeffreyense* at 20 IJ/insect and when 0 IJs/insect were applied. *Steinernema yirgalemense* at 60 IJs/insect was not significantly different from *S. jeffreyense* at 60 IJs/insect (98.3%), *S. yirgalemense* at 40 IJs/insect (95.0%) and *S. jeffreyense* at 40 IJs/insect (94.1%). *Steinernema yirgalemense* at 40 IJs/insect, *S. jeffreyense* at 40 IJs/insect, *S. yirgalemense* at 20 IJs/insect (88.3%) and *S. jeffreyense* at 20 IJs/insect (85.8%) gave *T. absoluta* larval mortality not significantly different from each other while it gave significantly higher (*p* < 0.05) mortality than the untreated *T. absoluta* larvae (Fig. 1).

### Leaf trial

#### EPN virulence

Analysis using a one-way ANOVA showed that there was a significant effect (*F*_(3, 36)_ = 25.34, *p* < 0.001) of the treatment on percentage mortality. *Steinernema yirgalemense* (58.9%) resulted in significantly higher (*p* < 0.05) *T. absoluta* larval mortality than *S. jeffreyense* (46.4%). The EPN concentrations gave 6.5, 34.6, 52.5 and 70.8% mortality for 0, 20, 40 and 60 IJs/insect, respectively. All the four EPN concentrations resulted in *T. absoluta* larval mortality that was significantly different (*p* < 0.05) from each other.

**Figure 2:**
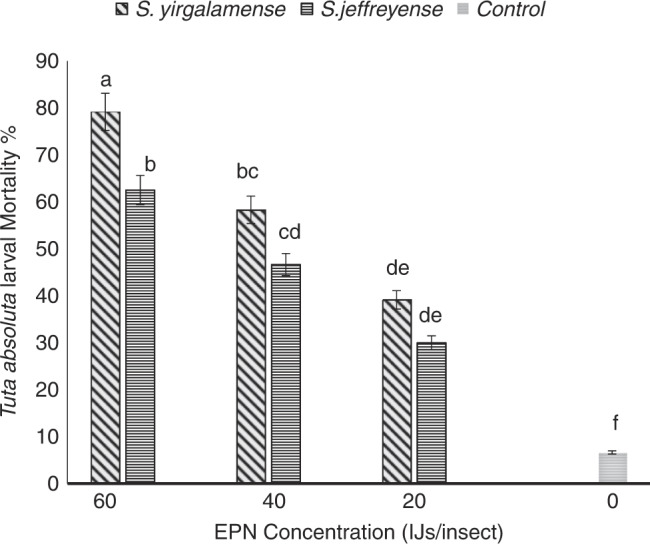
Mean percentage mortality (95% confidence level) of *Tuta absoluta* larvae inoculated with 0, 10, 20, and 40 IJs/insect of *Steinernema yirgalemense* and *Steinernema jeffreyense* (two-way ANOVA: *F*_(3, 28)_ = 17.50, *p* < 0.001) in a leaf trial. Different letters above the vertical bars indicate significant differences (*p* < 0.05).

There was a significant effect (F_(3, 28)_ = 17.50, *p* < 0.001) of the treatment on the percentage mortality. *Steinernema yirgalemense* at 60 IJs/insect (79.2%) provided significantly higher (*p* < 0.05) mortality of the *T. absoluta* larvae, than all the other combinations applied. *Steinernema jeffreyens* at 60 IJs/insect (62.5%) gave larval mortality that was not significantly different from *S. yirgalemense* at 40 IJs/insect (58.3%) but significantly different (*p* < 0.05) from the other combinations. *Steinernema yirgalemense* at 40 IJs/insect and *S. jeffreyense* at 40 IJs/insect (46.7%) gave larval mortality not significantly different from each other but different (*p* < 0.05) from the other combinations. *Steinernema jeffreyense* at 40 IJs/insect and *S. yirgalemense* at 20 IJs/insect (39.1%) gave mortality not different from each other but significantly different (*p* < 0.05) from the other combinations. *Steinernema jeffreyense* at 20 IJs/insect (30.0%) gave larval mortality not significantly different from *S. yirgalemense* at 20 IJs/insect but significantly different (*p* < 0.05) from the other combinations. The control gave *T. absoluta* larval mortality significantly lower (*p* < 0.05) compared to all the other combinations (Fig. 2).

## Discussion

Two EPN species, *S. yirgalemense* and *S. jeffreyense* isolated from sub-tropical environment, were used for the first time against *T. absoluta* larvae with successful control obtained for both species. Results from the current study are consistence with Van Damme et al. (2016), who reported that *S. carpocaspsae* and *S. bacteriophora* are also capable of killing *T. absoluta* larvae. Both EPN species recorded higher mortality rates, confirming the nematode’s potential to be used in an IPM program against *T. absoluta*. Husin (2017) also reported that three commercial EPN species were successful in killing the larvae and adults of *T. absoluta* after both growth stages were continuously exposed to the EPNs for 48 h. The author recorded 100% mortality when the EPNs were exposed to second, third and fourth larval instars while 96% was recorded when the first larval instar was exposed to the EPNs. In the current study, however, only the fourth larval instars were used resulting in 93% mortality in the laboratory bioassay and 58.8% when the EPNs where sprayed on a leaf infested with *T. absoluta*.

In another study by Batalla-Carrera et al. (2010), the third and fourth larval instars of *T. absoluta* were reported to be highly susceptible to continuous exposure to *S. carpocapsae*, *S. feltiae* and *H. bacteriophora* for up to 48 h. The same was reported by Mutegi et al. (2017) when two EPN species (*Heterorhabdities* sp. and *Steinernema karii*) were used to test their effect on *T. absoluta* mortality, with both studies reporting that EPNs were able to infest and kill *T. absoluta* larvae under laboratory conditions. In another study by Ngugi et al. (2018), indigenous EPN isolates (TK1, S86, 97, 75 and R52) from Kenya were screened for infectivity on 2nd and 3rd larval stages of *T. absoluta*. All the five of the indigenous EPNs were effective against *T. absoluta* larvae as they caused larval mortality and infective juveniles were recovered. They explained that the ability of EPN to infect and reproduce within the host pest ensures persistence and recycling ability in the natural environment. This is critical for effective pest control and commercial production efficiency of EPNs (Kalia et al., 2014; Ngugi et al., 2018).

In the larvae bioassay, there was no significant difference on mortality caused by the two different EPN species. Both EPN species had equal capability of infesting and killing the larvae when *T. absoluta* larvae were exposed on the filter paper. In the leaf bioassay, *S. yirgalemense* was more effective compared to *S. jeffreyense*. Host cues including the release of carbon dioxide and other odorants released by host attract or repel EPNs which normally cause directional movement in laboratory studies (Lewis et al., 1993; Grewal et al., 1994; Bal and Grewal, 2015). The nematodes, using the cues, needed to first find the larvae inside the leaf mines and different nematodes respond differently on carbon dioxide cues. In the current study, *S. yirgalemense* is smaller in size compared to *S. jeffreyense*. With the narrow natural openings on *T. absoluta* larvae, the natural openings are capable of reducing the invasion of IJs making it difficult for the nematodes to enter (Renn, 1998). Such results are in line with Bastidas et al. (2014) who observed that the size of the EPN species plays an irrefutable role in limiting penetration of host insects with larger EPNs less capable of invading smaller hosts.

Bal and Grewal (2015) reported that EPNs have to locate suitable hosts in order to complete their life cycle. Laboratory studies show differences in the host finding behavior of EPNs including cruisers which are active searchers, and ambushers which sit and wait foragers for the potential host (Lewis et al., 1992; Campbell and Gaugler, 1993; Bal and Grewal, 2015). The two EPNs used in the current showed differences in the way they located *T. absoluta* larvae in the leaf mines. A forager nematode actively moves through the soil or substrate to locate sedentary and slow-moving insects by detecting the carbon dioxide or other volatiles released by the insects (Campbell and Gaugler, 1997). *Steinernema jeffreyense* IJs were able to locate and infest find less *T. absoluta* larvae while *S. yirgalemense* may be an intermediate forager that employs both an ambush and a cruise foraging strategy which assisted in locating *T. absoluta* in the leaf mines (Campbell and Gaugler, 1997). Therefore, the intermediate forager *S. yirgalemense* is more effective at finding the larvae of *T. absoluta* inside the mines than the ambush forager *S. jeffreyense.*

The increase in mortality with an increase in concentration can be attributed to large number of EPNs and symbiotic bacteria released by the EPNs when they penetrate the larvae as reported by Eleftherianos et al. (2010). Mutegi et al. (2017) reported that *Heterorhabditis sp*. and *Steinernema karii* were able to kill *T. absoluta* larvae at various concentrations: 100, 300, and 500 IJs/ml. *Tuta absoluta* larvae mortality increased with increase in concentrations. The highest concentration (500 IJs/ml) achieved the highest mortality. Goudarzi et al. (2015) on a study of the effects of *H. bacteriophora* and *S. carpocapsae* as a biological control agents of *Agrotis segetum* (Lepidoptera) found that the highest larval mortality was achieved when EPNs were applied at a dose of 100 IJs/insect compared to 25, 50 and 75 IJs/insect. In another study by Dlamini et al. (2019), they reported that *S. yirgalemense, H. noenieputensis* and the exotic *S. feltiae* gave good control of banded fruit weevil (BFW), *Phlyctinus callosus* (Schönherr)(Coleoptera:Curculionidae) larvae at 100 IJs/insect.

The results on the leaf bioassay experiment showed that *S. yirgalemense* and *S. jeffreyense* were able to kill *T. absoluta* larvae on tomato leaves, regardless of their relative size. Batalla-Carrera et al. (2010), on the study of efﬁcacy of EPNs against *T. absoluta* in laboratory and greenhouse conditions, showed that EPNs are able to ﬁnd and kill larvae on tomato leaves, despite their relative position (inside or outside the tomato leaf). Mortality caused by nematodes at a rate of 60 IJs/cm^2^ varied from 76.3% by *H. bacteriophora* to 88.6% and 92% by *S. carpocapsae* and *S. feltiae*, respectively. The above results concurs with those reported by Gozel and Kasap (2015), who reported that EPNs are able to enter feeding canals in the leaves of tomatoes and kill *T. absoluta* larvae. Many larvae of *T. absoluta* died inside galleries, which show that IJs were able to find and infect them inside the mines.

The current results are not in agreement with those obtained by Sabry et al. (2016), who reported that *T. absoluta* mines per leaflets slightly decreased when *S. carpocapsae* was used alone against *T. absoluta*. The percentages of leaflets mines reduction in the first, second and third application were 10.5, 17.8 and 12.9%, respectively. The results showed that spinetoram and indoxacarb resulted in 99.3 and 80% mine reduction, respectively while *S. carpocapsae* alone was the least toxic. The results indicated that *S. carpocapsae* was not effective against *T. absoluta* due to the susceptibility of *S. carpocapsae* to some abiotic factors such as drought, light, UV radiation and high temperature. Also, the presence of the *T. absoluta* larvae in tunnels reduces the effectiveness of *S. carpocapsae*. In the current study, reduced mortality in the leaf bioassay was as a result of the fact that the EPNs were to look and find the insect in the mines while in the laboratory bioassay the *T. absoluta* larvae were exposed to the EPNs.

*Steinernema yirgalemense* at 60 IJ/insect was found to be more effective in controlling *T. absoluta* both in the laboratory and leaf bioassay. Such findings reveal that for the control of *T. absoluta* larvae in tomato leaves, *S. yirgalemense* would be more effective compared to *S. jeffreyense*. *Steinernema jeffreyense* is bigger in size compared to *S. yirgalemense*, making it to be easily limited by sieve plates present in opercules, stopping the nematode from penetration and causing more mortality (Ishibashi and Kondo, 1990). The smaller size of *T. absoluta* larvae coupled with smaller natural openings sizes hindered *S. jeffreyense* from entering through the normal infection routes or the larvae may produce smaller amounts of attractants such as carbon dioxide or kairomones, which makes it more difficult for nematodes to locate them (Husin, 2017).

If *S. jeffreyense* is the only nematode available, 40 IJs/insect is the maximum concentration that would be used to control *T. absoluta* larvae in leaf mines. This is because when the concentration of *S. jeffreyense* increases to 60 IJs/insect the same mortality of *T. absoluta* larvae is observed, hence increasing concentration will be a waste of the nematodes and resources. Van Damme et al. (2016) reported that mortality caused *H. bacteriophora*, *S. carpocapsae* and *S. feltiae* at a rate of 60 IJs/cm^2^ varied from 76.3, 88.6 and 92% for *H. bacteriophora*, *S. carpocapsae* and *S. feltiae*, respectively. This also confirms that different EPNs applied at the same rate can result in different mortality, showing the virulence of each EPN species. The same results were observed in the current study where *S. yirgalemense* caused higher mortality compared to *S. jeffreyense*.

## Conclusion

The two EPN species tested in the current study were effective in killing the larvae of *T. absoluta* under laboratory conditions regardless of their position and *S. yirgalemense* was more effective in killing *T. absoluta* larvae compared to *S. jeffreyense*. An increase in concentration resulted in an increase in mortality and among the evaluated nematodes concentrations, 60 IJs/insect was found to be more effective in killing *T. absoluta* larvae. The most effective nematode and EPN concentration combination to kill *T. absoluta* was 60 IJs/insect in both the lab and leaf bioassay. *Steinernema yirgalemense* at 60 IJ/insect was effective is recommended as it showed higher mortality rate. The study also showed the possibility of using the EPN species in IPM programs which reduce pollution, detrimental effects on the environmental, human health and reduce pesticide residues on tomato fruits while delaying the development of insect resistant to insecticides.
